# Machine learning approaches for predicting and validating mechanical properties of Mg rare earth alloys for light weight applications

**DOI:** 10.1080/14686996.2025.2449811

**Published:** 2025-01-31

**Authors:** Sandeep Jain, Ayan Bhowmik, Jaichan Lee

**Affiliations:** a School of Advanced Materials Science and Engineering, Sungkyunkwan University, Suwon, Republic of Korea; b Department of Materials Science and Engineering, Indian Institute of Technology Delhi, New Delhi, India

**Keywords:** Thermomechanical behavior, light weight Mg alloys, rare earth elements, machine learning

## Abstract

In this work, we have attempted to predict the mechanical behaviour of light weight Mg-based rare earth alloys fabricated through different mechanical and thermal processes. Our approach involves machine learning techniques across a range of different thermomechanical processes such as solution treatment, homogenization, extrusion and aging behaviour. The effectiveness of machine learning models is evaluated using performance metrics, including Coefficient of determination (R^2^), Mean Absolute Error (MAE) and Root Mean Square Error (RMSE). After modeling and selection of best model, the mechanical behaviour of new alloys was predicted in terms of ultimate tensile strength, yield strength and total elongation. The predicted results highlight the superior predictive accuracy of the K-Nearest Neighbors (KNN) machine learning model, demonstrating its better performance metrics compared with other machine learning approaches. This model has been found to predict the material properties with an effective evaluation matrix (R^2^ = 0.955, MAE = 3.4% and RMSE = 4.5%).

## IMPACT STATEMENT

This research introduces a machine learning approach for rapidly developing magnesium-based rare earth alloys, enhancing mechanical properties and optimizing design through a comprehensive set of parameters and revealing that KNN model exhibited superior performance.

## GRAPHICAL ABSTRACT

**Figure:**
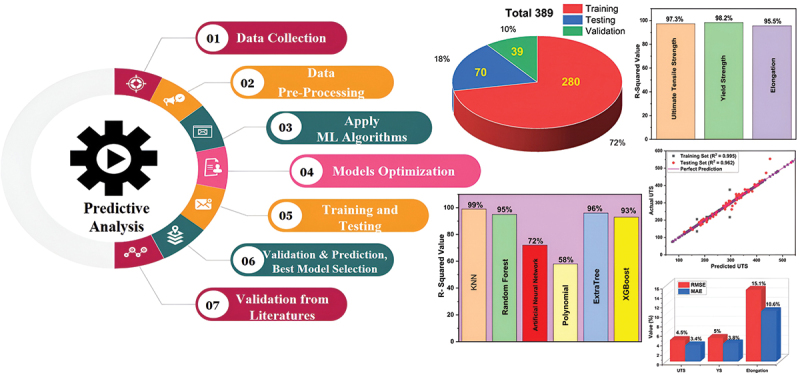

## 1. Introduction

**Figure 1.**
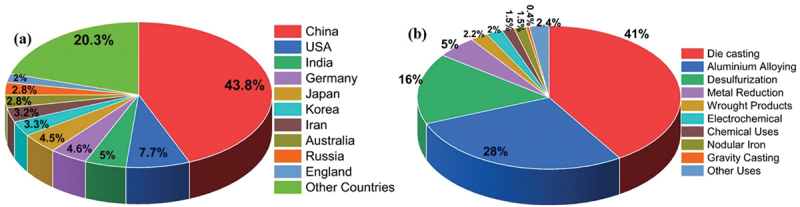
(a) Distribution of publications related to Mg and its alloys from different parts of the world (b) applications of Mg-alloys in different industries.

In the past few decades, there has been a growing need for materials that are both light weight and possess high strength [1,2]. This demand has led to extensive research on aluminum (Al) and magnesium (Mg) alloys, representing a noteworthy advancement in the creation of advanced materials applicable in various fields [3–6]. In Al and Mg alloys, Mg alloys are gaining prominence in the automotive and electronic industries because they stand out as the lightest metals among all structural materials [7–9]. Their exceptional properties, such as high specific strength, machinability etc. make them a subject of considerable interest in these industrial sectors [10]. Mg alloys are widely used in various applications and numerous research are also currently ongoing across the world [7,11,12]. The application and publications from different parts of world on Mg alloy are shown in Figure 1. Nevertheless, their utilization is constrained by issues related to insufficient strength and limited ductility. These challenges impose limitations on their viability for use in structural components of vehicles. Expanding the use of magnesium alloys necessitates finding ways to improve their properties [11]. Numerous approaches exist for enhancing the mechanical properties of Mg alloys such as addition of alloying elements [13,14], different mechanical and thermomechanical processing conditions [15,16] and different heat treatment conditions [17]. These require conducting long-term experiments through careful design of experiments and ensure the data is repeatable and reliable – all of this causes the product cost very high.

In practice, the exploration for an ideal precipitation microstructure tailored to a particular application is typically challenging, time-consuming, and expensive [18]. This is due to the necessity of delineating the optimal process conditions through activities such as heat treatments, microstructure characterization, and mechanical testing. An economical alternative is presented through the use of machine learning (ML) models [19–21], numerical simulations [22], ab initio modelling [23], etc. In recent times, ML has gained prominence as a potential tool for expediting the discovery of materials [24–26]. Numerous studies in the field of material science have extensively explored the applications of machine learning (ML), highlighting its potential to revolutionize the approach to studying materials [27–30]. Utilizing the capabilities of advanced computing and algorithms, researchers have been able to analyze extensive datasets comprising of diverse set of variables. This often involves evaluating the mechanical behavior of material as a function of thermomechanical process to understand and predict the mechanical behaviour of Al and Mg alloys [31–33]. With sufficient precision, ML algorithms and statistical analysis are used to glean insights from inputs, comprehend the underlying data structure and construct models that align with the given data. These ML algorithms have the capability to execute automated decision-making procedures and formulate predictions in a well manner.

It is widely acknowledged that the mechanical behavior of all metals and alloys is fundamentally connected to their composition, with this relationship being particularly significant in Mg alloys due to their sensitivity in mechanical and structural response to alloying additions. Consequently, altering or designing the alloy composition proves to be a viable approach for enhancing the overall properties of Mg alloys. Many researchers have suggested that Zn is a good choice to improve the room and high temperature tensile properties with some other minor elements like Nd, Y, Zr and Cu [26]. The use of rare earth elements (REEs) as alloying components in biodegradable metals has drawn considerable attention due to their unique atomic configuration and inherent properties. The rare earth alloying elements has a great effect on the mechanical behaviour of Mg alloy instead of common alloying elements [34]. The incorporation of zinc into magnesium-based materials is known to boost their strength by refining the grain structure and promoting precipitation strengthening. Cerium (Ce) enhances room temperature deformability, Neodymium (Nd) reduces corrosion rates and improves mechanical properties, and Yttrium (Y) boosts ultimate tensile strength and elongation of Mg-based alloys [35]. Gadolinium (Gd) significantly enhances the strength and creep resistance of magnesium alloys when exposed to high temperatures. Rare earth magnesium alloys possess distinctive properties compared to other metals, such as high specific strength, low thermal conductivity, excellent damping capacity and superior castability. A large number of researchers have reported the influence of rare earth elements in Mg alloy and informed that Mg-based rare earth alloys has better mechanical properties compared to common Mg alloys [36]. Therefore, a lot of combination of these alloying elements have been made to collect the dataset for mechanical behaviour of Mg rare earth alloys.

There are many types of thermomechanical processes to increase the mechanical behaviour of cast magnesium alloys such as solution treatment, homogenization, extrusion and aging at various temperatures. Many researchers have tried one or two options from the abovementioned processes to evaluate the mechanical behaviour of magnesium alloys. Suh et al. [37] reported the effect of extrusion process on the mechanical behaviour of Mg-Zn-Al alloys with the help of ML algorithms. Sarmah et al. [38] has reported the modelling approach for predicting the mechanical behaviour of age hardened Mg alloys. Kang et al. [26] have shown the effect of extrusion and heat treatment to optimize the mechanical properties of biogradable Mg-Zn-Y-Nd alloys. Österreicher et al. [39] presented a quantitative estimate of the mechanical properties of precipitation hardened Al-Mg-Si alloys. The examination of existing literatures indicates that maximum work has been done related to one or two parameters. But there is no study of Mg alloys to predict the mechanical behaviour by considering all mechanical and heat treatment process parameters and therein lies the necessity of this study.

Those abovementioned finding inspire us to forecast the mechanical properties, especially tensile properties of magnesium rare earth alloys by considering different combination of alloying elements and thermomechanical processes parameters. In the present work, the mechanical behaviour has been predicted by considering Mg, Zn, Y, Zr, Nd, Ce and Gd alloying elements and different thermomechanical processing as descriptors such as solution treatment, aging, homogenization at different temperatures and times as well as extrusion behaviour at different ratio and temperatures. One of the objectives of the present study is to validate the most appropriate model from literatures and open inaugurate a novel avenue for the design and development of Mg rare earth alloys for light weight applications.

## 2. Modelling approach

The most important step needed for machine learning is the collection of datasets according to the requirement to train the model. Therefore, in the present study, total 389 datasets have been used to predict the mechanical behaviour of magnesium alloys with different heat treatment processes and mechanical process. The used dataset was collected from different literatures [37,40–44] The dataset comprises information on seven rare earth elements (Mg, Zn, Y, Zr, Nd, Ce and Gd) used in various applications. The goal is to predict the mechanical behavior of Mg rare earth alloys, considering various descriptors such as Solution heat treatment temperature and time, Homogenization heat treatment temperature and time, Aging temperature and time and the Extrusion process with temperature and extrusion ratio. Solution and homogenization heat treatments significantly influence mechanical properties by dissolving intermetallic phases, reducing chemical segregation, and promoting a more uniform microstructure. Aging, also known as precipitation hardening, is crucial for determining mechanical properties, as varying aging temperatures and times enhance yield strength through the precipitation of fine, coherent particles that hinder dislocation movement. Additionally, extrusion refines grains and introduces a specific crystallographic texture, both of which play crucial roles in enhancing the strength and ductility of the alloy. In the case of magnesium alloys, the crystallographic texture induced by extrusion has a dominant effect on ductility, as it influences the slip system activity and the overall deformation behavior of the material. The mathematical relationships between these processes and mechanical properties are detailed in the supplementary file. The details of the collected dataset are shown in Table 1.

**Table 1.** Input and output parameters for the dataset used in the present study.

S.No.		Parameters	Range of Values
1	Input Parameters	Mg (wt%)	75.19–99.27
2		Zn (wt%)	0–15.17
3		Y (wt%)	0–18.14
4		Zr (wt%)	0–2
5		Nd (wt%)	0–4
6		Ce (wt%)	0–1.4
7		Gd (wt%)	0–20
8	Descriptors	Solution Treatment Temperature (T_sol_) (°C)	0–550
9		Solution Treatment Time (t_sol_) (hours)	0–48
10		Homogenization Temperature (T_hom_) (°C)	0–560
11		Homogenization Time (t_hom_) (hours)	0–48
12		Extrusion Temperature (T_extrusion_) (°C)	0–500
13		Extrusion Ratio (ER)	0–78.9
14		Aging Temperature (T_aging_) (K)	0–250
15		Aging Time (t_aging_) (hours)	0–126
16	Output Parameters	UTS (MPa)	75–575
17		YS (MPa)	20.9–532.9
18		Elongation (%)	0.5–37.59

In this study, different Machine Learning (ML) algorithms were conducted for training with the aim of predicting mechanical behaviour by considering different aspect of dataset. The spectrum of algorithms investigated includes (i) Multilayer Perceptron (MLP), (ii) Gradient Boosting (XGB/XGBoost), (iii) Random Forest (RF), (iv) Extra Tree (ET), (v) K-Nearest Neighbors (KNN) and (vi) Polynomial regression models.

The choice of these ML models is driven by their unique strengths in addressing the challenges of predicting mechanical properties under diverse processing conditions. For instance, Decision Tree is employed for non-linear relationships and the model excels in handling feature interactions; while Random Forest, as an ensemble method, mitigates overfitting and copes well with non-linearity and high-dimensional data; KNN operates without assumptions about data distribution and is flexible with non-linear patterns; XGB is a specialized gradient boosting library designed for swift and efficient computation, emphasizing rapid performance. XGB often outperforms other implementations and has become a popular choice in various machine learning competitions; Extra Tree, with its advantages of reducing overfitting, fast training and adeptness in handling noisy data, is also considered; Polynomial regression introduces polynomial terms to capture non-linear relationships. In essence, each ML approach brings its distinct advantages to the table, contributing to a comprehensive exploration of the predictive capabilities for mechanical properties under diverse processing conditions.

**Figure 2.**
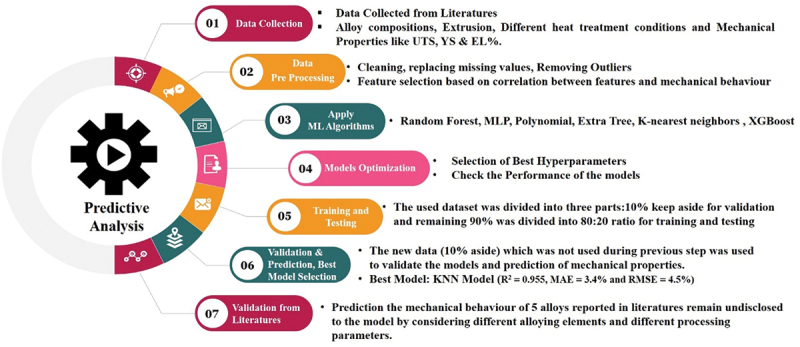
Different steps involved in the present study to predict the mechanical behaviour.

After an initial evaluation of their performance, the top four machine learning models were determined for predicting mechanical behaviour based on their performance. The models which are showing more than 80% R^2^ value, have been selected for further prediction. After validating all top four models, one most appropriate model was chosen to predict the mechanical behaviour of reported alloys at various parameters and suggest the importance of different processing parameters to obtain better mechanical behaviour of Mg alloy. Evaluation metrics, including R-squared value, Mean Absolute Error (MAE) and Root Mean Square Error (RMSE), were used to evaluate the precision and effectiveness of the selected models. The basic structure which has been followed in the current study is shown in Figure 2. The figure shows a chart of the different steps involved in the present study from collection of data to prediction behaviour of new alloy based on literature to suggest the various optimized parameters for the mechanical behaviour of Mg alloys.

This modeling approach is used to predict the all three mechanical properties simultaneously. Performance of all models was checked based on multi-objective performance data. After that, the predictions of UTS, YS and % EL were shown one by one to show the predictability in a clear way.

## 3. Results and discussion

### 3.1. Correlation between different features

The dataset used for predicting the mechanical behavior of Mg rare earth alloys encompasses variations in alloying elements under different thermomechanical processing parameters. A total of 389 entries were gathered for this dataset, serving as the foundation for predicting mechanical behavior through the application of various ML techniques. All six models as mentioned previously – MLP, XGB, RF, ET, KNN and PR were explored in this study.

The parameters of each model were optimized through a grid search algorithm to fine-tune the hyperparameters. The analysis was conducted using the Python module ‘scikit-learn 1.4.1. post1’. During model development, 10% of the data was kept aside, and the remaining 90% was further divided into a ratio of 80:20 for training and testing the models. The data was normalized using the ‘min-max scaler’ from the used module before being fed into the models. After developing the model, we then denormalized the predicted data to obtain values in the original data format. The goal of assessing these machine learning techniques across diverse processing scenarios is to ascertain the most effective method for predicting mechanical properties.

Heat maps are essential tools in machine learning, providing visual representations of feature and label distributions in datasets. They facilitate the identification of correlations between features, aiding in the detection of redundant or highly correlated variables that can optimize model performance. The visualization often includes expressing feature importance through correlation coefficients, such as the widely used Pearson correlation coefficient. The statistical analysis utilized the ‘pandas 2.0.3’ package for computation. The correlation between any two features is assessed by Equation 1, which generates a correlation value between −1 and + 1, representing both the strength and direction of their relationship [45]:

(1) $$r_{\mathrm{xy}}=\mathrm{Corr}\left(X,Y\right)=\frac{\mathrm{cov}\left(X,Y\right)}{\sigma_{x}\sigma_{y}}=\frac{\sum_{i=1}^{n}\left(X_{i}-\overset{ˉ}{X}\right)\left(Y_{i}-\overset{ˉ}{Y}\right)}{\sigma_{x}\sigma_{y}}$$

**Figure 3.**
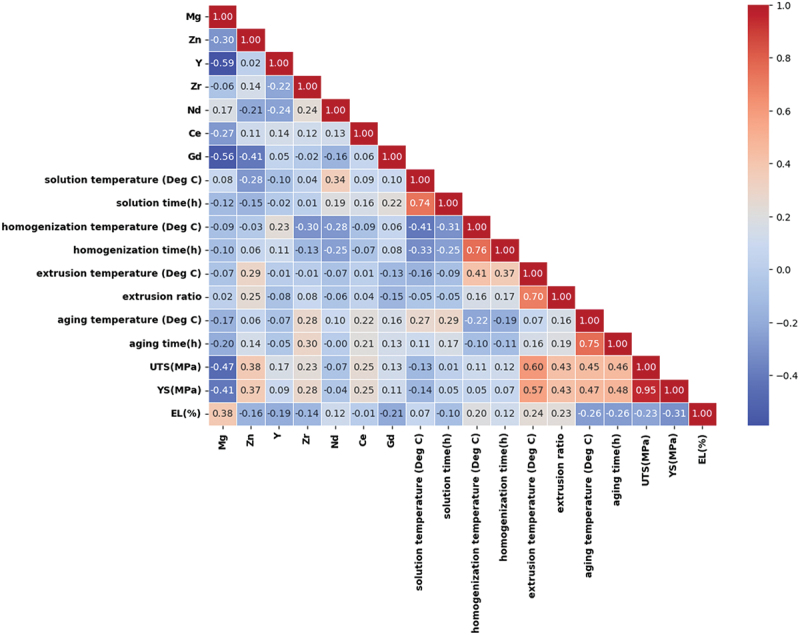
Heat map depicting correlations among all features.

In this equation, ‘n’ represents the sample size, $\overset{ˉ}{x}$ and $\overset{ˉ}{x}$ denote the mean values of the input features x and y, and σ_x_ and σ_y_ are representing their respective standard deviations. The heatmap provides a visual representation of how flow stress correlates with all features present in the dataset. Figure 3 specifically highlights essential features that have a notable impact on mechanical properties.

It is clear from Figure 3 that out of all processing features, two features (extrusion and aging behaviour) show very good correlation with mechanical properties and other two features are also showing the acceptable correlation with mechanical properties. In Figure 3, the heat map shows a weak but positive correlation between the rare earth content and both UTS and YS, with correlation values slightly above 0.2. This suggests that rare earth elements do improve strength properties, but their effect is modest. Other factors in conjunction with the rare earth element, such as alloy composition and processing parameters, likely play a significant role in determining the overall mechanical properties. Thus, while the weak correlation does not rule out strengthening effects, it highlights the complexity of the influence of rare earth elements in these alloys. Therefore, all dataset has been considered to predict the mechanical behaviour without discounting any features.

### 3.2. Prediction of mechanical behaviour using different models

#### 3.2.1. Selection of best hyperparameters

Choosing the most appropriate hyperparameters for a machine learning model is essential to attain the best possible performance. Hyperparameters refer to parameters that are established prior to the commencement of the training process and are not subject to adjustment during the training phase. They essentially control the learning process of the model. The performance of a model is heavily influenced by the hyperparameters chosen. Tuning hyperparameters can lead to better performance metrics of each model. Hyperparameter tuning is performed using grid search techniques algorithms. The process entails experimenting with various combinations of hyperparameter values and assessing the model’s performance to identify the optimal set of hyperparameters that yield the best results for a given task. The optimized best hyperparameters of each model have been selected, and these parameters are given in Table 2.

**Table 2.** Optimized hyperparameters of all models.

S.No.	Models	Best Parameters
1	KNN	{‘n_neighbors’: 5, ‘p’: 1, ‘weights’: ‘distance’}
2	RF	{‘max_depth’: 10, ‘min_samples_leaf’: 1, ‘min_samples_split’: 2, ‘n_estimators’: 150}
3	MLP	{‘activation’: ‘relu’, ‘alpha’: 0.01, ‘hidden_layer_sizes’: (100, 50, 25), ‘learning_rate’: ‘constant’}
4	Polynomial	{‘linearregression__fit_intercept’: True, ‘polynomialfeatures__degree’: 1}
5	Extra Tree	{‘max_depth’: 20, ‘min_samples_leaf’: 1, ‘min_samples_split’: 5, ‘n_estimators’: 150}
6	XGBoost	{‘colsample_bytree’: 0.8, ‘learning_rate’: 0.1, ‘max_depth’: 5, ‘min_child_weight’: 5, ‘n_estimators’: 50, ‘subsample’: 0.8}

#### 3.2.2. Models performance

After completing the training of the model, the performance of the trained model was checked on the base of testing data. The performance or accuracy of any model can be decided by three indices such as R^2^ value, MAE and RMSE.

R^2^ (R-squared) quantifies the predictability of the dependent variable (target) based on the independent variables (features), with values ranging from 0 to 1. A perfect fit is represented by an R^2^ of 1, and a higher R^2^ signifies an improved model fit to the data.

(2) $$R^{2}=1-\frac{\sum_{i=1}^{N}{\left(\sigma_{e_{i}}-\sigma_{p_{i}}\right)}^{2}}{\sum_{i=1}^{N}{\left(\sigma_{e_{i}}-{\overset{ˉ}{\sigma }}_{e_{i}}\right)}^{2}}$$

MAE measures the mean absolute difference between predicted and actual values, offering interpretability and equal treatment of errors without emphasizing outliers. It is less sensitive to extreme values compared to RMSE.

(3) $$\mathrm{MAE}=\frac{1}{N}\overset{N}{\underset{i=1}{\sum }}|\sigma_{e_{i}}-\sigma_{p_{i}}|$$

RMSE assigns greater importance to significant errors through the squared term, offering an average error magnitude with increased penalty for larger discrepancies. Its sensitivity to outliers makes it more susceptible to influence from extreme data values.

(4) $$\mathrm{RMSE}=\sqrt{\frac{1}{N}\overset{N}{\underset{i=1}{\sum }}{\left(\sigma_{e_{i}}-\sigma_{p_{i}}\right)}^{2}}$$

Here, $\sigma_{e_{i}}$ is the representation of experimental values, $\sigma_{p_{i}}$ is the representation of predicted values, ${\overset{ˉ}{\sigma }}_{e_{i}}$ is the representation of the average of experimental values for each observation i respectively and N is the representation of the total number of samples used during machine learning.

**Figure 4.**
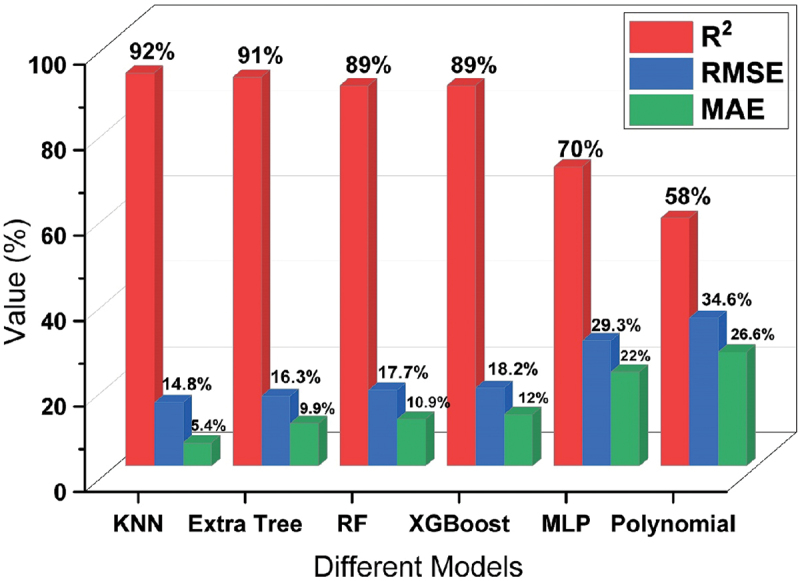
Overall performance of all models based on testing data.

**Figure 5.**
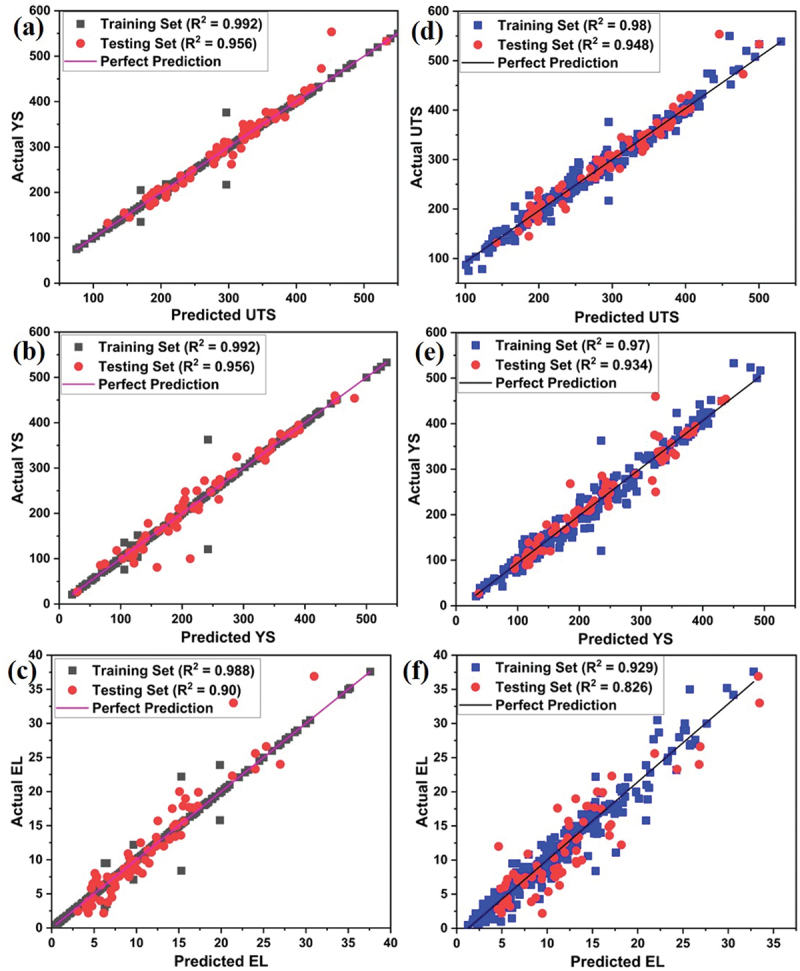
Performance of models on training and testing data for (a, b, c) KNN model (d, e, f) RF model for UTS, YS and % elongation respectively.

The summary of the performance of all trained models is shown in Figure 4 wherein it is observed that MLP and Polynomial regression models are not suited for the prediction of different mechanical properties of Mg rare earth alloys. The performance of all 6 models on the base of training and testing data are shown in Figure 5 and supplementary file (Figure S1, S2 & S3). Figure 5 Showing that the KNN model is performing well during training as well as testing compare to all other models to predict the UTS, YS and %EL.

In Figure 5, some differences between the actual and predicted values are noted, which can be attributed to several factors. First, machine learning models may not always predict individual data points accurately due to inherent variability in the data while they are effective in capturing overall trends. Second, experimental or measurement noise, as well as the complexity of the system, including non-linear interactions not fully represented in the model, can contribute to these deviations. Finally, the limited feature set used in the model may also fail to capture all relevant variables influencing the outcomes. Despite these discrepancies, the model successfully captures the general trends, and the validation remains robust.

#### 3.2.3. Model validation

**Figure 6.**
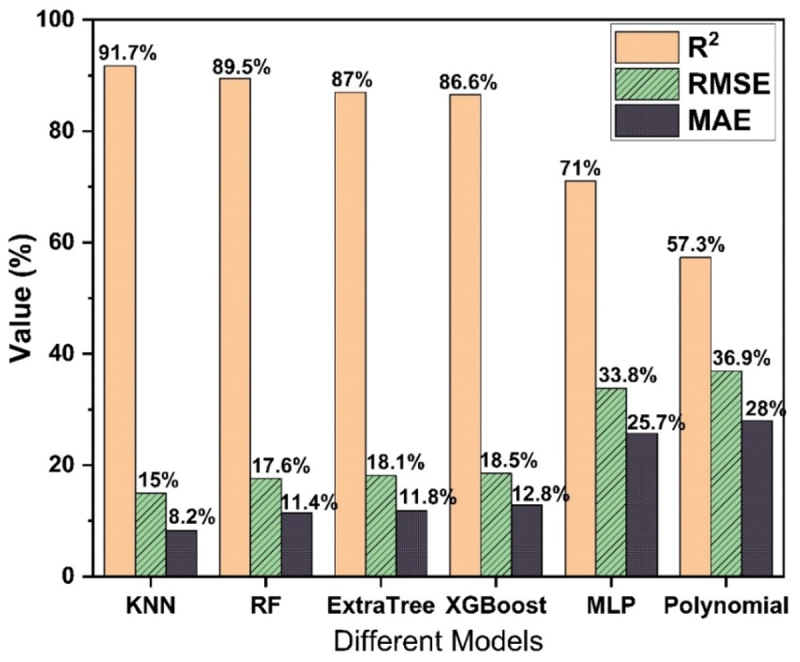
Overall performance of all models based on validation data.

The validation process plays a pivotal role in the evolution of machine learning models. Validation is used to assess how well a model is expected to perform on data that was not used during training. This process helps estimate the model’s ability to generalize, indicating its error rate on new, unfamiliar data. This is crucial because the primary objective of a machine learning model is to generate precise predictions when presented with new, real-world data. Using the test data for validation doesn’t provide an independent assessment of the model’s generalization performance because the model has already seen the test data during the evaluation phase. Hence, a reserved 10% of the data was designated for validation purposes and remained untouched during the training of all models. The overall performance of all 6 models have been evaluated on validation data as depicted in Figure 6. Figures 4 & 6 show that the top 4 models such as Random Forest, KNN, XGBoosting and Extra Tree regression are performing well. Therefore, these 4 models are used to predict the mechanical properties separately and to select one most appropriate model to predict the mechanical properties and suggest the best processing parameters for design the Mg rare earth alloys.

##### 3.2.3.1. Ultimate tensile strength

**Figure 7.**
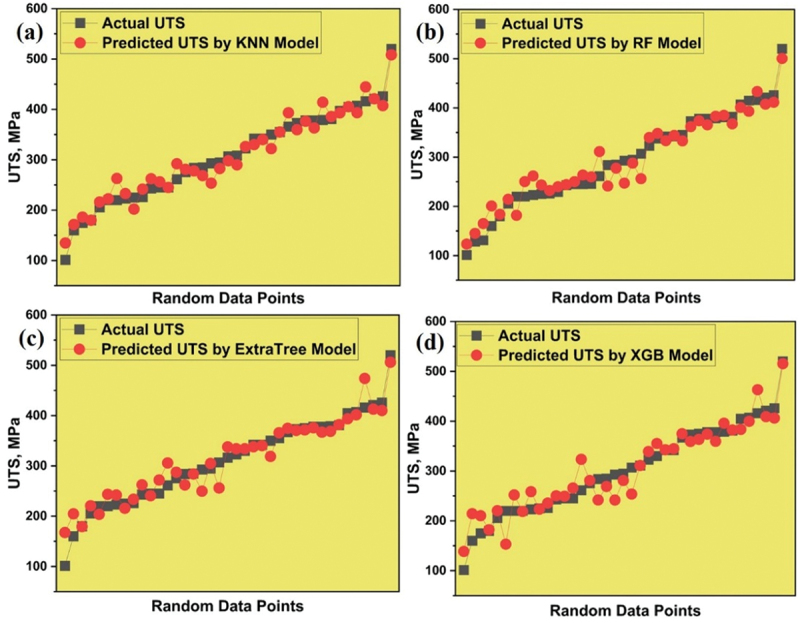
Variation between predicted and actual UTS for (a) KNN (b) RF (c) ET and (d) XGB models.

The selected models were employed to forecast the UTS of magnesium alloy using novel data, which the trained model had not encountered during its training and testing phases. The disparity between the predicted UTS and the actual or experimental UTS is visually represented in Figure 7(a–d). Analysis of the graph indicates that the data points projected by the KNN model closely align with the actual data points, surpassing the performance of all other models which clearly indicates that the KNN model stands out as the most accurate and reliable among them. The comparison of actual and predicted UTS from 4 models is shown in supplementary data in Figure S4(a).

**Figure 8.**
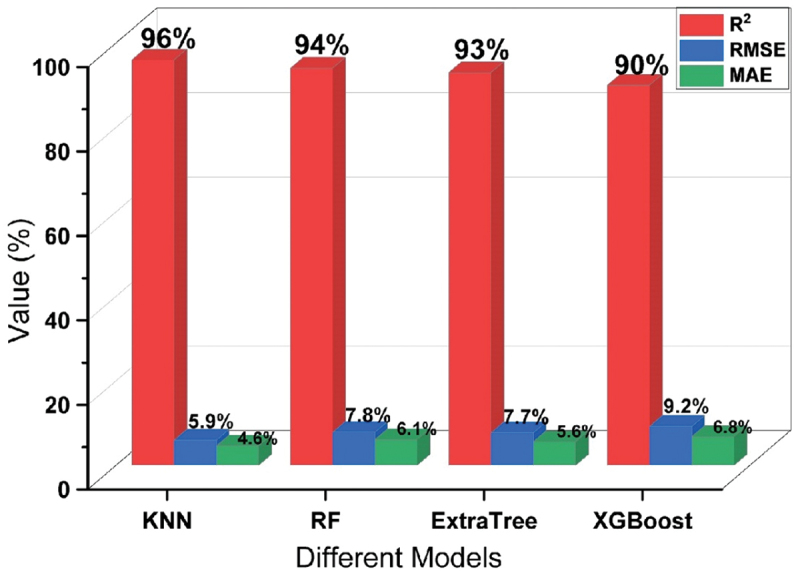
Performance of all models for UTS prediction.

The graphical representation in Figure 8 illustrates the performance of selected models based on their distinct evaluation metrics. As depicted in Figure 8, it is evident that the KNN model emerges as the most suitable choice for accurately predicting the UTS of different Mg-based rare earth alloys.

##### 3.2.3.2. Yield strength

**Figure 9.**
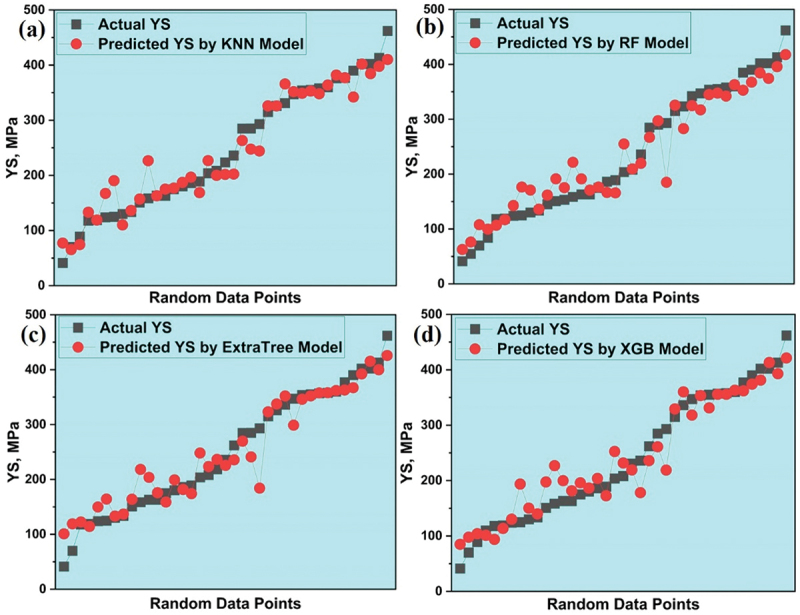
Variation between predicted and actual YS for (a) KNN (b) RF (c) ET and (d) XGB models.

The chosen models were utilized to predict the yield strength (YS) of various Mg rare earth alloys using previously unseen data, ensuring that the models were evaluated on novel information not encountered during their training and testing phases. The divergence between the predicted YS through various models and the actual or experimental YS is visually depicted in Figure 9(a–d). A thorough examination of the plots reveals that the data points projected by the KNN model closely coincide with the actual data points, outperforming all other models which underscores the superior accuracy and reliability of the KNN model, establishing it as the most effective among the considered models. The comparison of actual and predicted YS from 4 models is shown in supplementary data in Figure S4(b).

**Figure 10.**
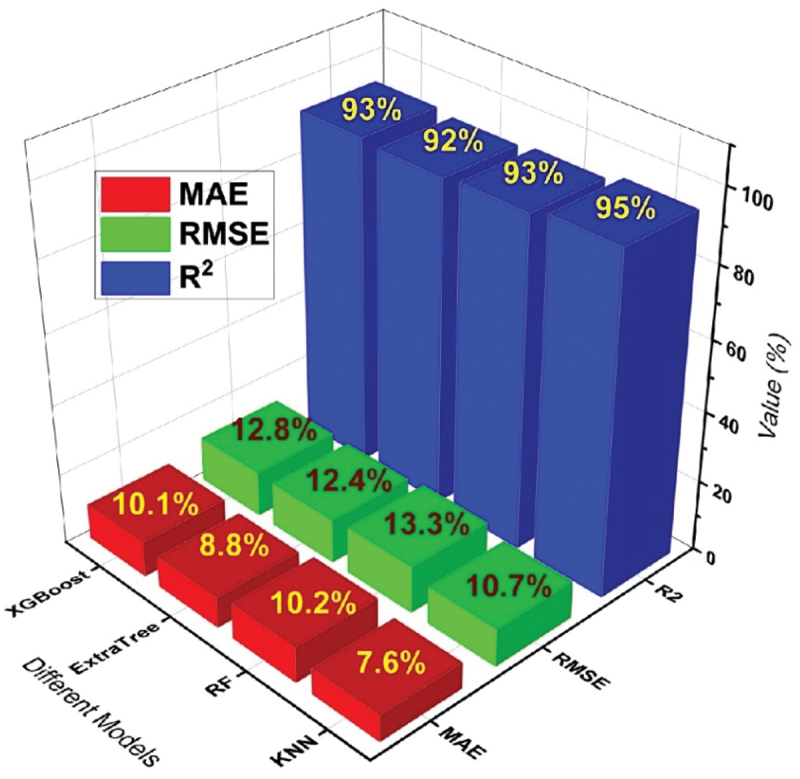
Performance of all models for YS prediction.

The visual depiction in Figure 10 showcases the performance of chosen models through their unique evaluation metrics. It is clearly observed in the figure that the KNN model stands out as the optimal selection for precisely forecasting the yield strength (YS) of various Mg-based rare earth alloys.

##### 3.2.3.3. Elongation

**Figure 11.**
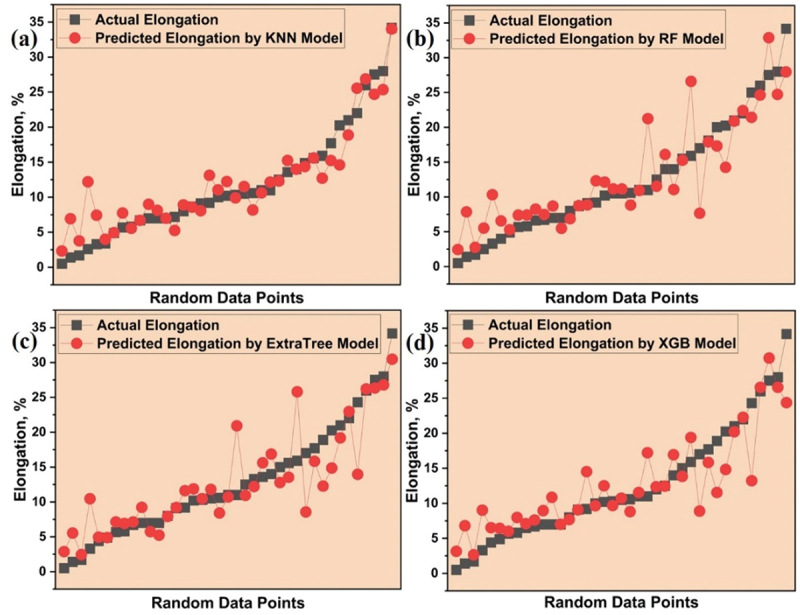
Variation between predicted and actual elongation for all (a) KNN (b) RF (c) ET and (d) XGB models.

The selected models were employed to forecast the elongation (EL) of Mg alloys similarly using previously unseen data, ensuring a rigorous evaluation on novel information not encountered during their training and testing phases. The disparity between the predicted EL and the actual or experimental EL is visually represented in Figure 11(a–d). A meticulous analysis of the graph reveals a remarkable alignment between the data points projected by the KNN model and the actual data points, superseding the performance of all other models which demonstrates superior accuracy and reliability, solidifying its position as the most effective among the considered models. The comparison of actual and predicted EL from 4 models is shown in supplementary data in Figure S4(c).

**Figure 12.**
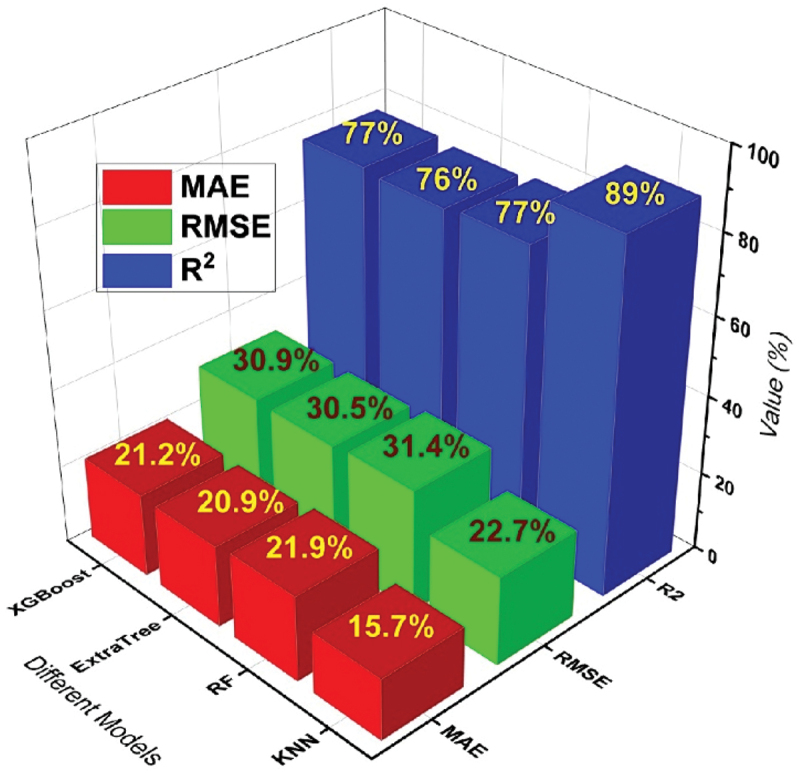
Performance of all selected models for elongation prediction.

The visual representation presented in Figure 12 highlights the performance of selected models based on their distinct evaluation metrics. As evident from the figure, the KNN model distinguishes itself as the preferred choice for accurately predicting the elongation (EL) of a range of Mg-based rare earth alloys selected.

The performance of top 4 models for the prediction of UTS, YS and EL% are given in Table 3 with all evaluation matrices.

**Table 3.** Performance of models based on new data.

S.No.	Models	UTS (MPa)			YS (MPa)			Elongation (%)		
		R^2^	RMSE	MAE (%)	R^2^	RMSE	MAE (%)	R^2^	RMSE	MAE (%)
1	KNN	0.96	5.95	4.6	0.95	10.7	7.6	0.89	22.7	15.7
2	RF	0.94	7.8	6.1	0.93	13.3	10.2	0.77	31.4	21.9
3	Extra Tree	0.93	7.7	5.6	0.92	12.4	8.8	0.76	30.5	20.9
4	XGBoost	0.90	9.2	6.8	0.93	12.8	10.1	0.77	30.9	21.2

### 3.3. Prediction of mechanical behaviour based on literatures

**Figure 13.**
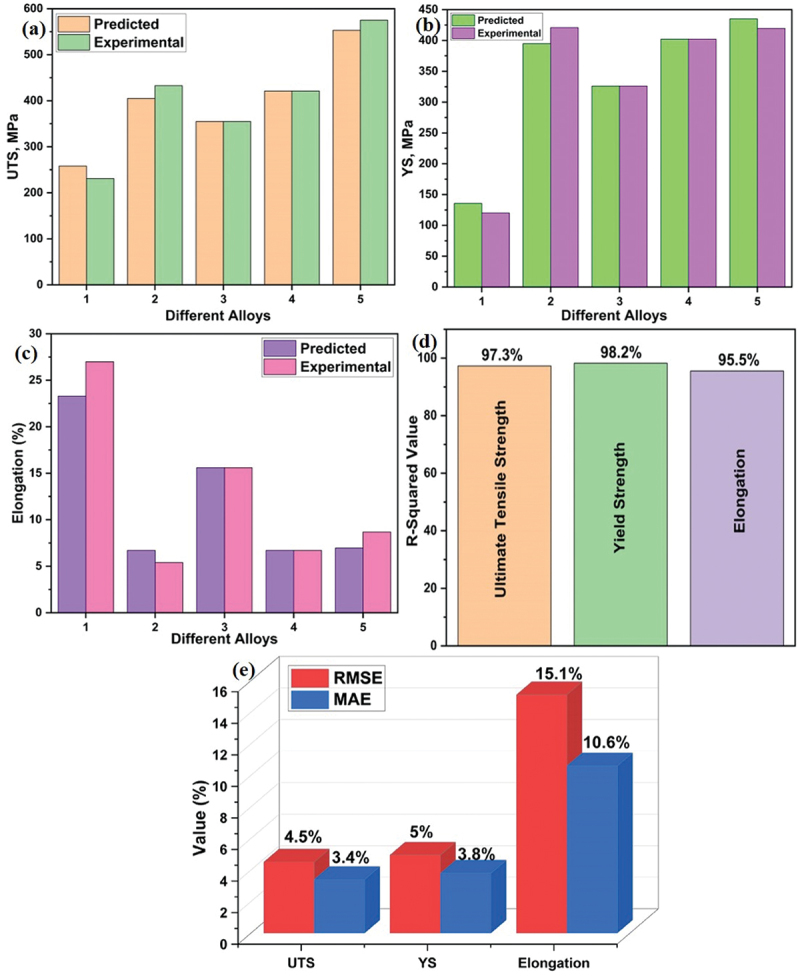
Comparison of KNN model for the prediction of (a) UTS (b) YS (c) elongation and performance of KNN model (d) R^2^ value (e) RMSE and MAE.

After getting validation performance of top four models, the most appropriate model, KNN model, was chosen to predict the mechanical behaviour of five reported alloys in literatures remaining undisclosed to the model by considering different alloying elements and different processing parameters. Five alloys were meticulously chosen, each emphasizing a distinct processing parameter, including extrusion or solution treatment (ST), extrusion and aging, and an encompassing approach involving all processing parameters in the dataset features. To assess the reliability of the model more comprehensively, the mechanical properties of an alloy selected based on two processing parameters (only extrusion, only aging as well as extrusion and aging) were forecasted by systematically varying the parameter values. Consequently, these five alloys collectively represent a comprehensive exploration of all conceivable variations in processing parameters, providing valuable insights into the predictive capabilities of the model. The prediction of mechanical properties from KNN model with its performance is shown in Table 4. The performance of KNN model for the prediction of new alloys are given in Figure 13. Extrusion parameters exhibit the best prediction compared to the other parameters.

**Table 4.** Validation of KNN model by predicting mechanical properties for 5 alloys.

S.No.	Alloys	Thermomechanical Processing Parameters						UTS (MPa)		YS (MPa)		EL (%)		Ref.
		T_sol_	t_sol_	T_extrusion_	ER	T_aging_	t_aging_	Pred.	Exp.	Pred.	Exp.	Pred.	Exp.	
1	Mg-2Zn-0.5Zr-0.5Nd	450	5	390	64	0	0	258.2	231	135.5	120	23.3	27	[44]
2	Mg-3Y–0.5Zr-12Gd	0	0	0	0	230	4	405.2	432.9	395	420.9	6.7	5.4	
3	Mg-5.97Zn-0.47Zr	0	0	320	18	200	12	355	355	326	326	15.6	15.6	[46]
4	Mg-6.23Zn-0.49Zr-1.54Nd	0	0	290	18	0	0	421	421	402	402	6.7	6.7	[47]
5	Mg-8Zn-0.7Zr-7.9Y–1.9Nd-1.1Ce-2Gd	239	2.4	297.6	9	192.5	64.7	553	575	435	419.4	6.95	8.68	[44]

## 4. Conclusions

This research quantitatively explores the mechanical properties of Mg-based alloys through machine learning technique. The results provide valuable insights into the application of ML for predicting mechanical properties in magnesium alloys and identifying essential variables for the design of light weight structural metals. The primary outcomes are summarized as follows:

1. Different ML algorithms have been tested to predict the mechanical properties of Mg-based rare earth alloys by considering different thermomechanical processing conditions according to the requirement.
2. The validation of these models with new data has been highly effective, demonstrating excellent performance in accurately predicting mechanical properties of Mg-based rare earth alloys.
3. The KNN (K-nearest neighbor) model proved to be the most reliable and robust algorithm for predicting mechanical (tensile) properties of Mg-based rare earth alloys.
4. The second most robust algorithm identified in the study was the Random Forest and ExtraTree, demonstrating the least likelihood of overfitting and minimal disparity between train and test scores. The findings suggest that these models also hold promise as an effective algorithm for capturing patterns related to composition, processing, structure and property in Mg-based rare earth alloys. Further enhancement may be achieved by incorporating additional training data.
5. The tensile mechanical properties of five alloys have been predicted using best KNN model, and the analysis shows a good predictability with high correlation (R^2^ = 0.955) and very little error (root mean square error, RMSE = 4.5%).
6. These findings bear considerable implications for the design of advanced high-performance Mg-based rare earth alloys with potential applications across various industries through high throughput machine learning–based approach. It is believed that this will result in considerable reduction in time and financial resource requirements by significantly eliminating complexities in experiment design and cutting down experimental rigours.

## Supplementary Material

Supplemental Material

## Data Availability

Data will be made available on request.
