# Supplementary material for: Machine learning approaches for predicting and validating mechanical properties of Mg rare earth alloys for light weight applications
Source: Sci Technol Adv Mater. 2025 Jan 31;26(1):2449811. doi: 10.1080/14686996.2025.2449811 (PMC11792136; doi:10.1080/14686996.2025.2449811)
Supplement: Supplemental Material [file TSTA_A_2449811_SM3933.docx]

**Supplementary File**

**Machine Learning Approaches for Predicting and Validating Mechanical Properties of Mg Alloys for Light weight applications**

Sandeep Jain^a,b#^, Ayan Bhowmik^b^, Jaichan Lee^a#^

***^a^*** School of Advanced Materials Science and Engineering, Sungkyunkwan University, Suwon, 16419, Republic of Korea

*^b^ Department of Materials Science and Engineering, Indian Institute of Technology Delhi, India*

**Results and Discussion**


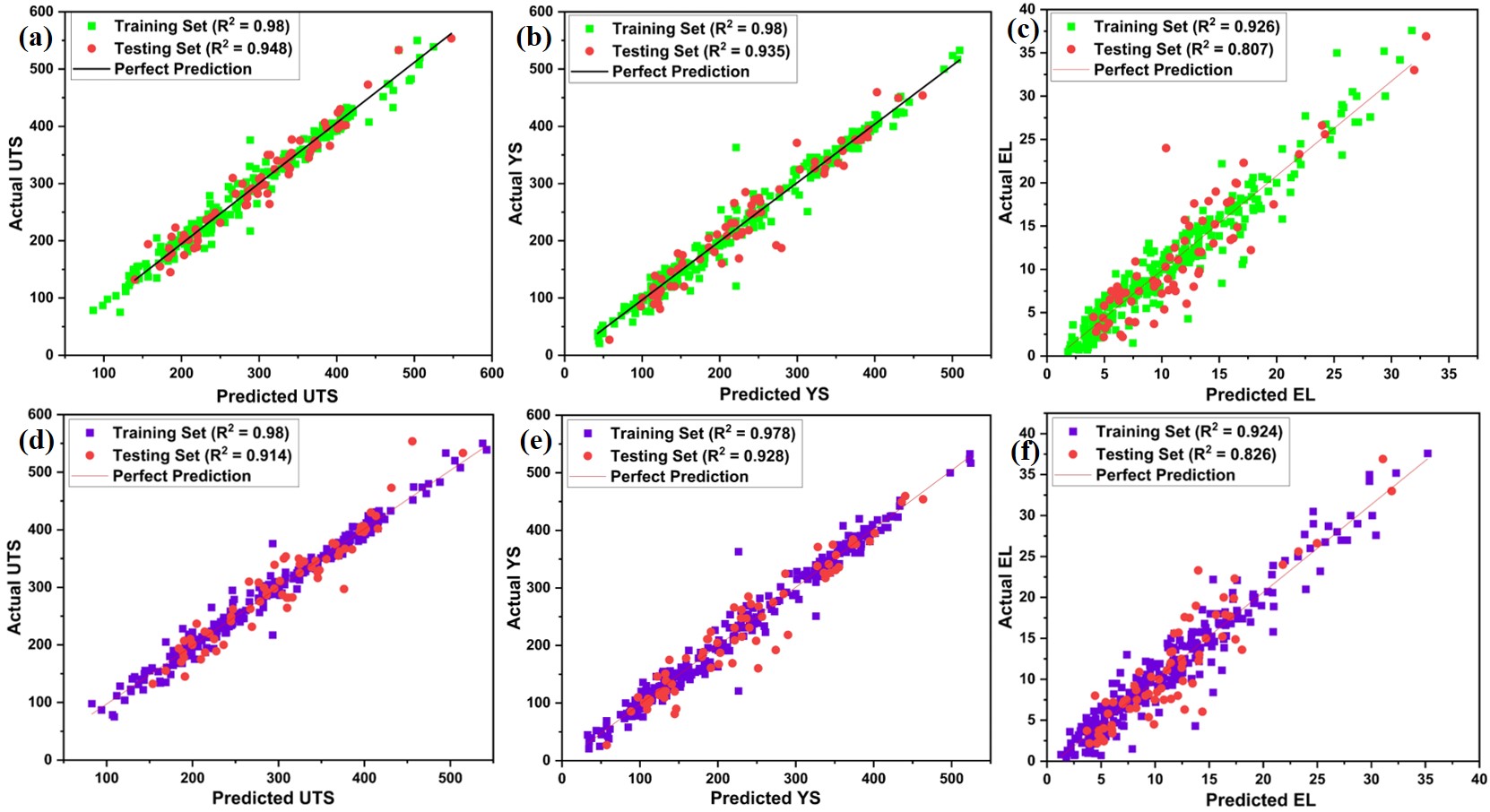


**Fig.S1** Performance of Models on training and testing data for (a, b, c) ExtraTree model (d, e, f) XGB model for UTS, YS and % Elongation respectively

Fig. S2 is showing the performance of MLP and Polynomial models based on their best hyperparameters during training and testing session. It is clear from Fig.S2 that Polynomial is showing the worst performance to predict the mechanical behaviour of Mg based alloys among all used models.


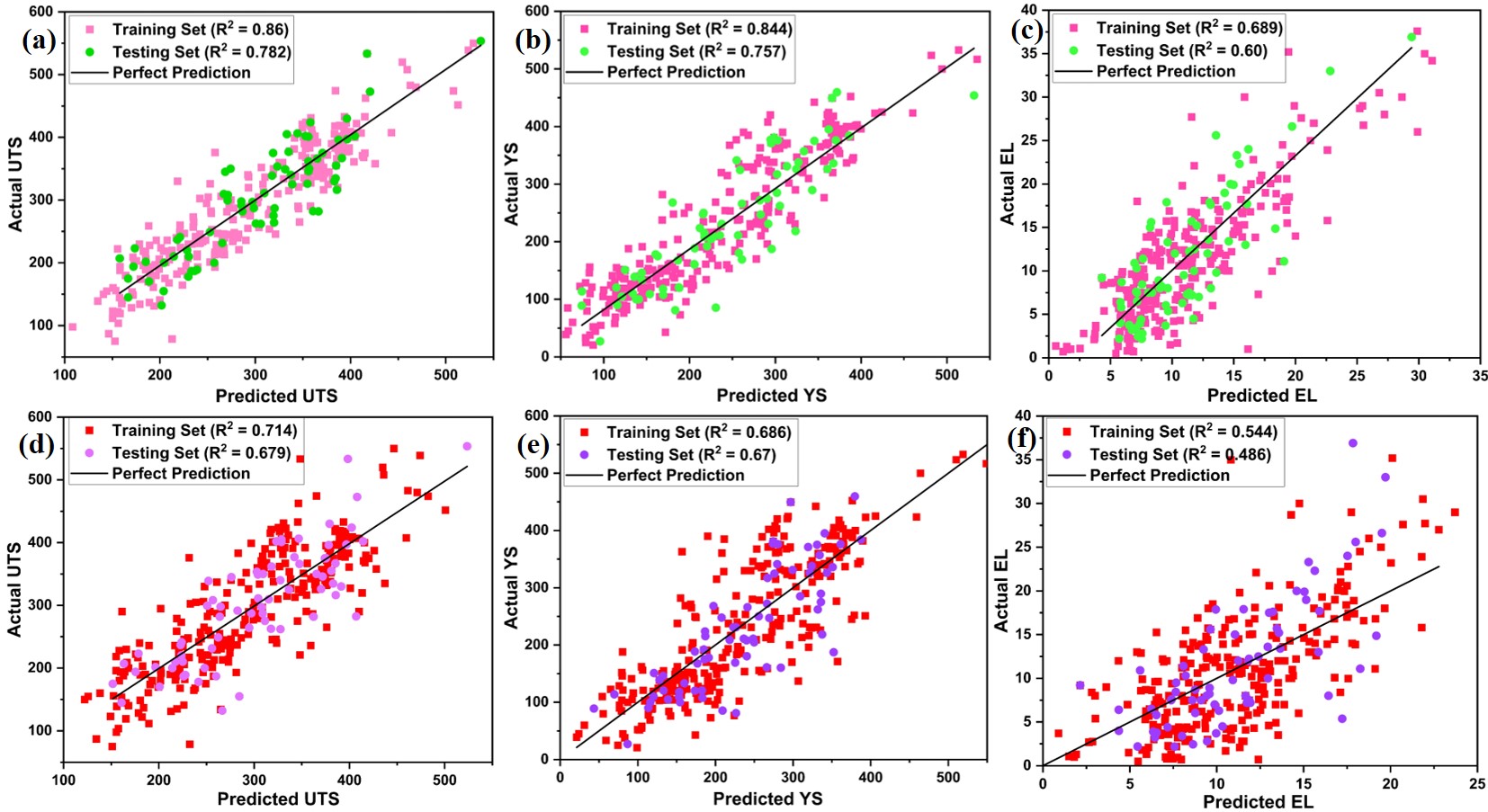


**Fig.S2** Performance of Models on training and testing data for (a, b, c) MLP model (d, e, f) Polynomial model for UTS, YS and % Elongation respectively

Fig. S3 is showing the performance and comparison of all 6 models for the prediction of UTS, YS and % EL during training and testing mode. It is clear from Fig. S3 that KNN is performing well in all conditions in respect of R^2^, RMSE as well as MAE.


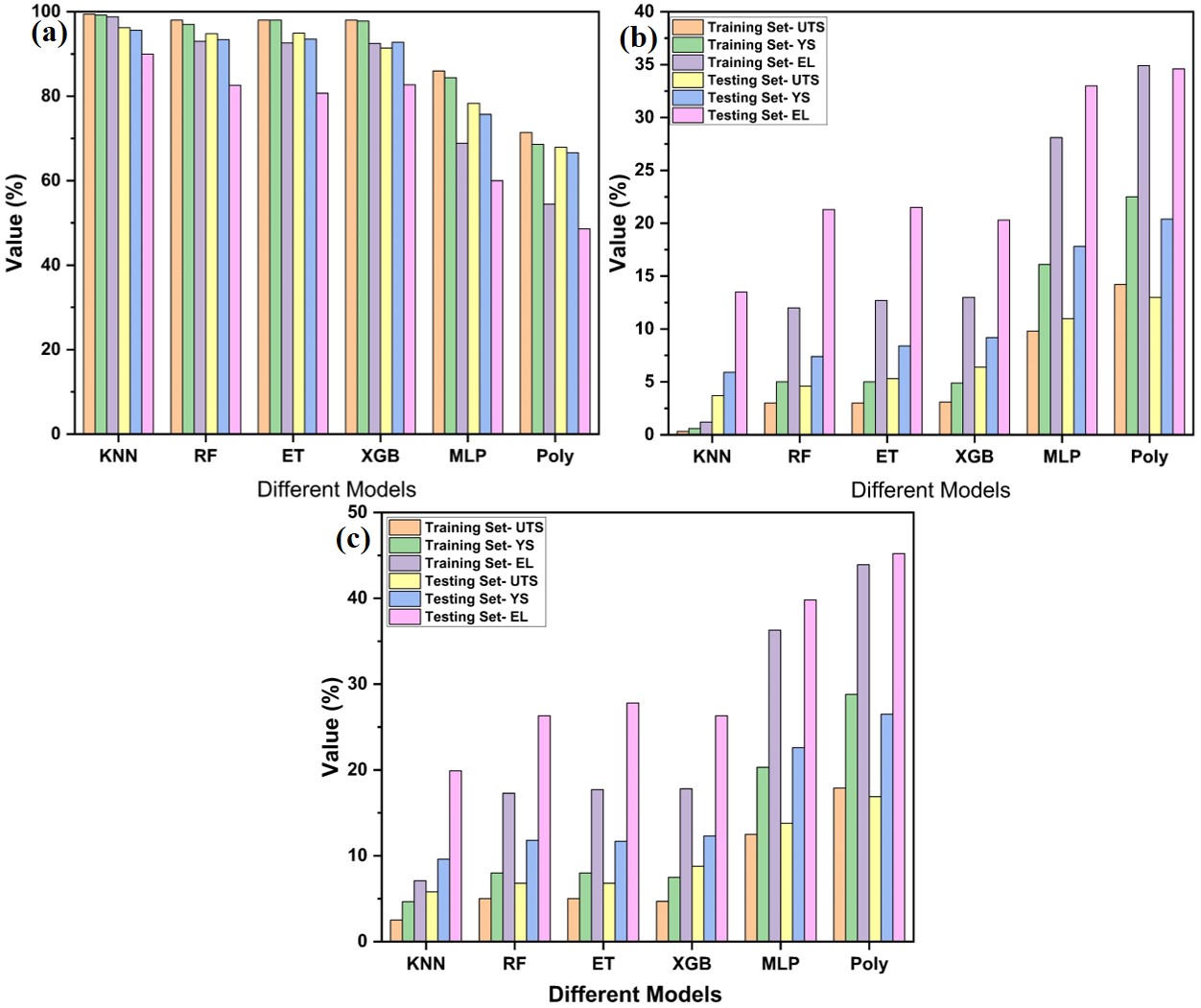


**Fig.S3** Performance of all 6 Models on training and testing data by their evaluating matrices (a) R^2^ value (b) RMSE (C) MAE for the prediction of UTS, YS and % Elongation respectively

Fig. S4 is showing the performance and comparison of top 4 models used in the present study. It is clear from Fig. S4 that among top 4 models, KNN is performing in well manner to predict the UTS, YS and % EL.


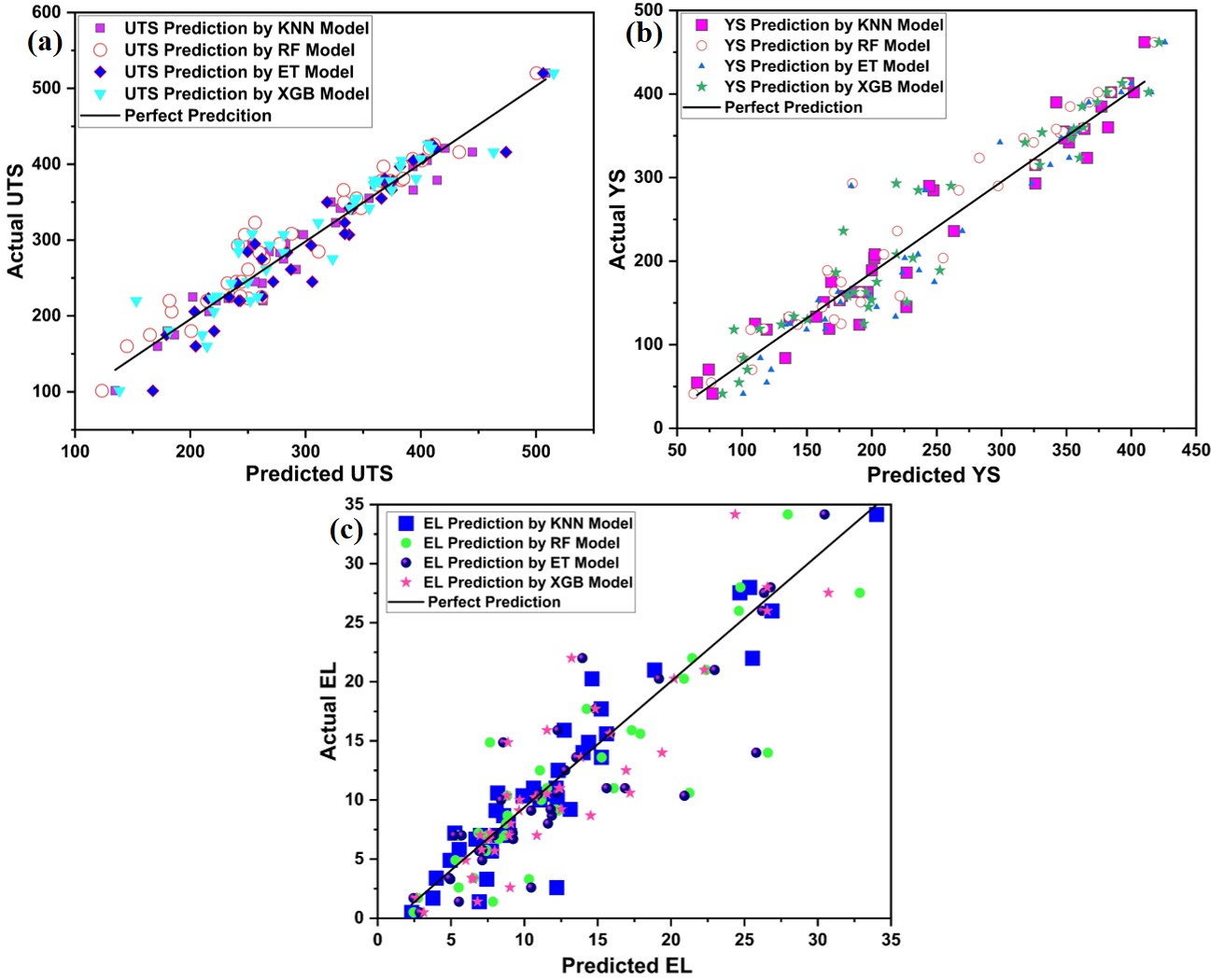


**Fig. S4** Comparison of Actual and Predicted mechanical properties from top 4 models (a) UTS Prediction (b) YS Prediction (c) EL Prediction
